# Surgical outcomes following nerve transfers in upper brachial plexus injuries

**DOI:** 10.4103/0970-0358.59272

**Published:** 2009

**Authors:** P. S. Bhandari, L. P. Sadhotra, P. Bhargava, A. S. Bath, M. K. Mukherjee, Tejinder Bhatti, Sanjay Maurya

**Affiliations:** Department of Plastic Surgery, Armed Forces Medical College & Command Hospital (SC), Pune - 40, India

**Keywords:** Nerve transfers, root avulsions, upper brachial plexus injury

## Abstract

**Background:**

Brachial plexus injuries represent devastating injuries with a poor prognosis. Neurolysis, nerve repair, nerve grafts, nerve transfer, functioning free-muscle transfer and pedicle muscle transfer are the main surgical procedures for treating these injuries. Among these, nerve transfer or neurotization is mainly indicated in root avulsion injury.

**Materials and Methods:**

We analysed the results of various neurotization techniques in 20 patients (age group 20-41 years, mean 25.7 years) in terms of denervation time, recovery time and functional results. The inclusion criteria for the study included irreparable injuries to the upper roots of brachial plexus (C5, C6 and C7 roots in various combinations), surgery within 10 months of injury and a minimum follow-up period of 18 months. The average denervation period was 4.2 months. Shoulder functions were restored by transfer of spinal accessory nerve to suprascapular nerve (19 patients), and phrenic nerve to suprascapular nerve (1 patient). In 11 patients, axillary nerve was also neurotized using different donors - radial nerve branch to the long head triceps (7 patients), intercostal nerves (2 patients), and phrenic nerve with nerve graft (2 patients). Elbow flexion was restored by transfer of ulnar nerve motor fascicle to the motor branch of biceps (4 patients), both ulnar and median nerve motor fascicles to the biceps and brachialis motor nerves (10 patients), spinal accessory nerve to musculocutaneous nerve with an intervening sural nerve graft (1 patient), intercostal nerves (3rd, 4th and 5th) to musculocutaneous nerve (4 patients) and phrenic nerve to musculocutaneous nerve with an intervening graft (1 patient).

**Results:**

Motor and sensory recovery was assessed according to Medical Research Council (MRC) Scoring system. In shoulder abduction, five patients scored M4 and three patients M3+. Fair results were obtained in remaining 12 patients. The achieved abduction averaged 95 degrees (range, 50 - 170 degrees). Eight patients scored M4 power in elbow flexion and assessed as excellent results. Good results (M3+) were obtained in seven patients. Five patients had fair results (M2+ to M3).

## INTRODUCTION

Upper brachial plexus injuries (C5, C6 with or without C7) present typically with lack of shoulder and elbow functions. Involvement of C7 spinal root may result in an additional weakness of elbow extensors and long extensors to the wrist and fingers. Nerve transfers remain viable option in the rehabilitation of these patients if the proximal roots are either avulsed or heavily scarred. A variety of donor nerves exist as a source for neurotization. Some of the more common neurotization sources include the spinal accessory nerve,[1,2] phrenic nerve,[3,4] medial pectoral nerve[5] and the intercostal nerves.[6,7] More recently, the use of a fascicle of a functioning ulnar or median nerve (Oberlin transfer) in patients with intact C8 and T1 has allowed a rapid and powerful return of elbow flexion.[8] The purpose of this study was to assess the outcomes of different neurotization techniques in the restoration of shoulder and elbow functions in patients with upper brachial plexus injuries.

## MATERIAL AND METHODS

Between February 2006 and April 2007, a total of 20 patients with upper brachial plexus lesions underwent surgical exploration and reconstruction of the brachial plexus. The three inclusion criteria in this study were irreparable injuries to the upper spinal roots, surgery within 10 months of injury and a minimum follow up period of 18 months. The term irreparable was used where spinal nerve roots were not considered suitable as donor nerves either due to avulsion injury or extensive fibrosis. The clinical examination included testing of motor power in upper extremity muscles using the British Medical Research Council Grading System [Table 1].

**Table 1:** Medical Research Council Grading System (MRC)

*Observation*	*Muscle grade*
No contraction	0
Flicker or trace of contraction	1
Active movement, with gravity eliminated	2
Active movements against gravity	3
Active movements against gravity and resistance	4
Normal power	5

All the patients underwent electromyography and 3D MR myelography using Siemens magnetom 1.5 Tesla equipment in which 3D data were reconstructed with 1 mm sections in axial, coronal and sagittal planes. The diagnostic accuracy was 74% when 3D MRI reports were compared with intraoperative findings.

Preoperative and postoperative video assessments of all the patients were carried out. The average shoulder abduction was measured against gravity, while the average shoulder external rotation was measured with the arm flexed next to the trunk and fully internally rotated.

### Surgical Technique

Under general anaesthesia, patient was placed in the supine position and the brachial plexus was explored through an incision starting along the posterior border of lower part sternocleidomastoid muscle and continuing above and parallel to the clavicle.

The patient's anaesthesia was maintained with short-acting muscle relaxants. A nerve stimulator was used at 0.5, 1.0 and 2.0 mA to identify the motor branches throughout the surgical exploration. The upper brachial plexus spinal nerves are generally present in the space between the anterior and middle scalene muscles. Their absence suggested root avulsions. This was correlated with the MRI myelography, which revealed the characteristic pseudomeningoceles in the presence of root avulsions.

The suprascapular nerve was located along the lateral aspect of the upper trunk. Often the proximal end of the suprascapular nerve was involved in the upper trunk neuroma. To identify the spinal accessory nerve, the anterior border of the trapezius muscle was located 2--3 cm above the clavicle. The fascia over the trapezius muscle was incised and detached from the anterior surface of the muscle. The deep cervical fascia was opened to expose the accessory nerve and its branches. The accessory nerve was dissected and sectioned as distally as possible. The most proximal and prominent branch was always identified and preserved. The suprascapular nerve was located in the vicinity and a direct coaptation was possible in all the cases. The phrenic nerve was located on the anterior surface of scalenus anterior muscle and identified by its vertical course and contractions of diaphragm on electrical stimulation. It was dissected distally and then divided and moved laterally for transfer.

Infraclavicular plexus was explored through an incision just medial to the deltopecteral groove and extending into the inner arm. Exposure of the cords and their terminal branches usually needed the division of pectoralis major and minor muscles. For the identification of posterior cord and the axillary nerve, the axillary artery was taped and pulled aside. The proximal part of axillary nerve was usually found near the inferior border of pectoralis minor muscle, where it branched out of the posterior cord. A semicircular incision was extended from the wound at the anterior border of the axilla on to the infraareolar region to gain access to the intercostal nerves. The deep central branches of the third, fourth and fifth intercostal nerves were used for transfer to musculocutaneous nerve. Oberlin transfers (partial transfers of the ulnar and median motor branches) were made through a longitudinal incision on the anteromedial aspect of upper arm. The musculocutaneous nerve was identified after it traversed the coracobrachialis muscle. The motor branch to biceps was usually seen at an average distance of 130 mm from the acromion. The nerve to the brachialis muscle was found at an average of 193 mm below the acromion.

The ulnar nerve was identified at the same level, and a longitudinal epineurotomy was made. One or two ulnar nerve fascicles, carrying motor fibres to the flexor carpi ulnaris (confirmed by electrical stimulation), were minimally dissected, sectioned and coapted to the biceps motor branch with 10-0 nylon suture. Fascicles of the median nerve that innervated the flexor carpi radialis, flexor digitorum superficialis or palmaris longus were identified and coapted with the motor branch to the brachialis. Again a tension-free nerve anastomosis was ensured. For the transfer of a motor branch to long-head triceps to the axillary nerve, patient was placed in semilateral position with upper arm over the thorax. An oblique incision was made along the posterior border of deltoid. Axillary nerve was identified in the quadrilateral space, bounded above by the teres minor muscle, below by the teres major muscle, laterally by the humerus and medially by the long head of triceps muscle. After emerging from the quadrilateral space, the axillary nerve gives branches to teres minor muscle and then divides into 1-3 anterior branch (es) and one posterior branch. The anterior branch or branches provide major motor supply to the deltoid. This branch or branches were dissected intraneurally as proximal as possible and transected. Through the inferior part of incision, the long and lateral heads of triceps muscle were separated and the radial nerve visualized in the triangular space. The motor branch to long head of triceps is usually given off at a distance of 90 mm from the angle of acromion. This branch was sectioned as distally as possible and then flipped 180° to be coapted to the anterior branch or branches of the axillary nerve. In all cases, nerve dissections were done under 4× loupe magnifications, and nerve coaptations were made under the operating microscope with 10-0 nylon sutures.

Transfer of spinal accessory and phrenic nerves to the musculocutaneous nerve always requires an intervening sural nerve graft. Phrenic nerve could be transferred directly to supracapular nerve. However, its transfer to axillary nerve required an intervening sural nerve graft.

Postoperatively, the flexed arm was strapped to the chest for a period of 3 weeks. After that gradually increasing passive exercises were begun in the shoulder and elbow joints. Paralysed muscles were subjected to electrical stimulation till M3 power was achieved.

The postoperative results were assessed as poor, fair, good and excellent as per classification proposed by Terzis *et al.*[9] depending on the muscle grade achieved [Table 2]. Various nerve transfers performed are shown in Table 3.

**Table 2:** Assessment scale used for postoperative functional evaluation (Terzis *et al.*)[9]

*Results*	*Muscle grade*
Poor	M0 to M2
Fair	M2+ to M3
Good	M3+ to M4
Excellent	M4 to M5

**Table 3:** Nerve transfer performed

*Patient no.*	*Intra-operative findings*	*Shoulder*	*Elbow*
1.	C5,C6 and C7 roots avulsed	SAN --- SSN	ICN - MCN
2.	C5,C6 and C7 root avulsions	SAN --- SSN	ICN---MCN
		PhN--ng---AXN	
3.	C5 root visibly intact but scarred with no response on electrical stimulation, C6 root avulsed	SAN ---- SSN	OB I and II
4.	C5,C6 roots scarred, weak contractions of deep cervical muscles on electrical testing	SAN --- SSN	OBI and II
		LHT --- AXN	
5.	C5,C6 and C7 roots avulsed	PhN ---- SSN	SAN -- ng-- MCN
6.	C5,C6 roots avulsed	SAN --- SSN	OB I
7.	C5,C6 and C7 roots avulsed	SAN ---- SSN	PhN -- ng -- MCN
8.	C5,C6 roots fibrosed, weak response on electrical stimulation	SAN --- SSN	OB I and II
		LHT ---- AXN	
9.	C5,C6 roots avulsed	SAN --- SSN	OB I
10.	C5,C6 roots scarred, weak response on electrical stimulation	SAN --- SSN	OB I and II
		LHT -- AXN	
11.	C5,C6 roots fibrosed, no response on electrical stimulation	SAN --- SSN	OB I
		LHT --- AXN	
12.	C5 visibly intact but scarred, C6 root avulsed. No response on electrical stimulation of C5 root	SAN --- SSN	
		LHT -- AXN	OB I and II
13.	C5,C6 and C7 roots avulsed	SAN --- SSN	ICN - MCN
		PhN -- ng- AXN	
14.	C5,C6 roots avulsed	SAN --- SSN	OB I and II
15.	C5,C6 roots visibly intact but extensively scarred, weak contractions of deep cervical muscles on electrical stimulation	SAN --- SSN	OB I and II
		LHT -- AXN	
16.	C5,C6 and C7 roots avulsed	SAN --- SSN	ICN - MCN
17.	C5,C6 and C7 roots avulsed	SAN --- SSN	
		ICN -- AXN	OB I and II
18.	C5,C6 roots avulsed	SAN --- SSN	OB I
19.	C5,C6 and C7 roots avulsed	SAN --- SSN	
		ICN -- AXN	OB I and II
20.	C5,C6 roots avulsed	SAN --- SSN	OB I and II
		LHT -- AXN	

SAN, spinal accessory nerve; SSN, suprascapular nerve; LHT, nerve to long head of triceps; PhN, phrenic nerve; AXN, axillary nerve; ICN, intercostal nerve; OB, Oberlin; ng, nerve graft.

All patients were evaluated at 3-month intervals. Range of movements were noted with goniometry. Sensory evaluation was made by measuring the 2-point discrimination at the pulp of index and little finger in the patients subjected to Oberlin transfers. British Medical Research Council Scoring System was used to evaluate the strength of elbow flexion, extension and shoulder abduction, ranging from 0 (no evidence of contractility) to 5 (complete range of motion against gravity with full resistance).

## RESULTS

**Figure 1:**
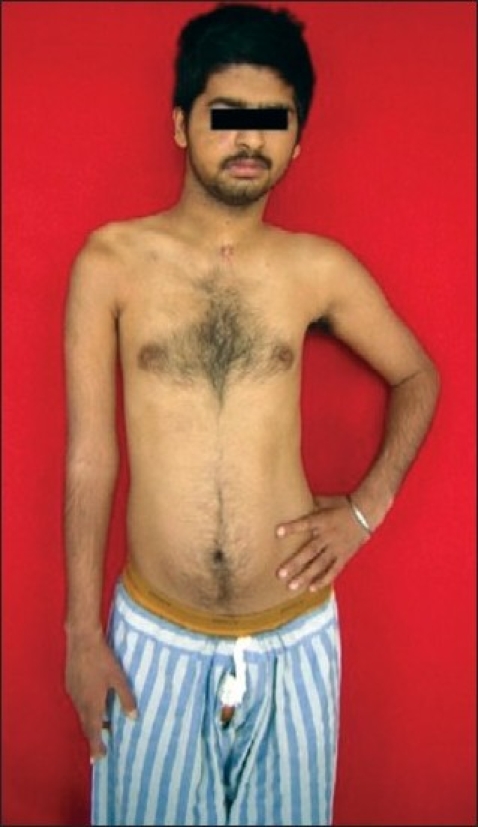
Right-sided upper brachial plexus (C5,C6,C7) palsy

**Figure 2:**
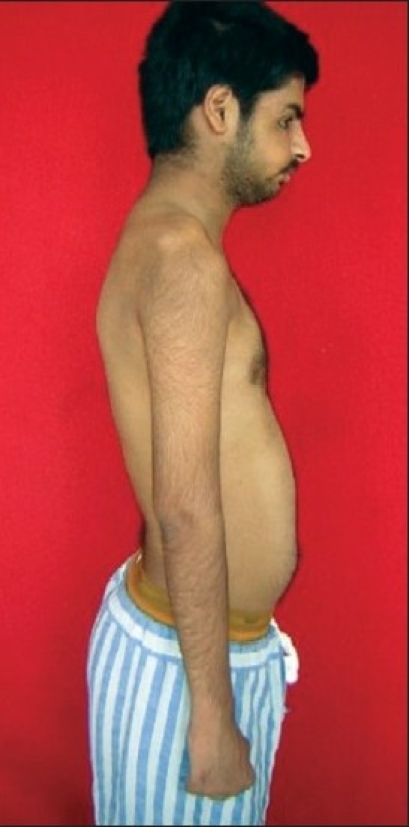
Marked wasting of shoulder and arm muscles

**Figure 3:**
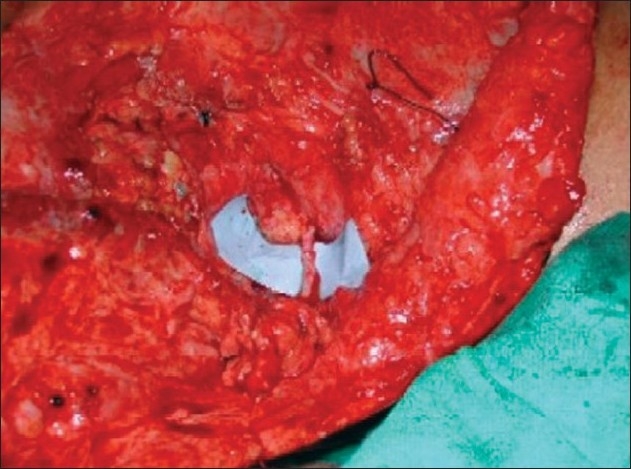
Transfer of spinal accessory nerve to suprascapular nerve.

**Figure 4:**
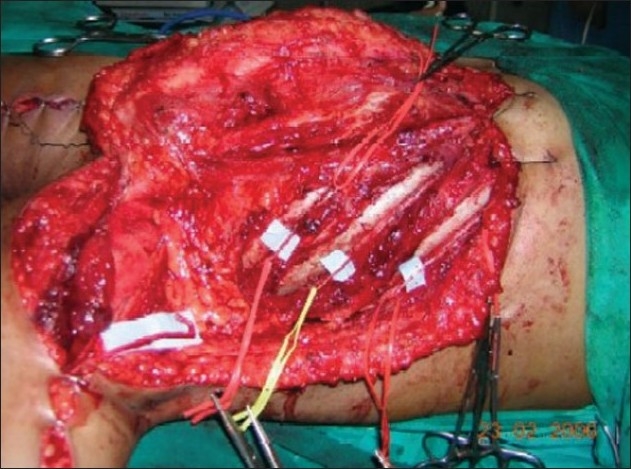
Dissection of 3rd, 4th, 5th intercostal and musculocutaneous nerves

**Figure 5:**
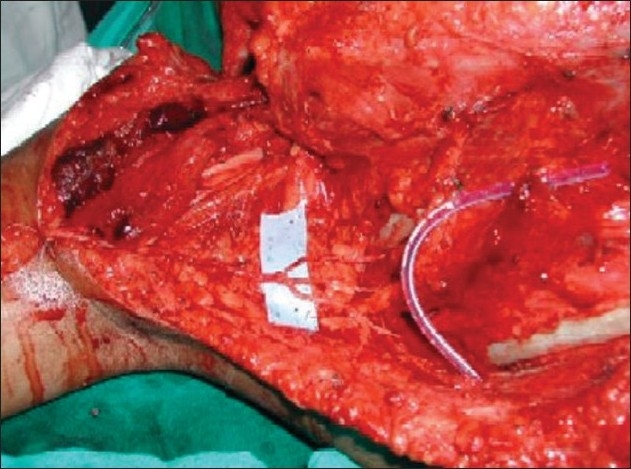
Three intercostal nerves have been transferred to the lateral part of musculocutaneous nerve

**Figure 6:**
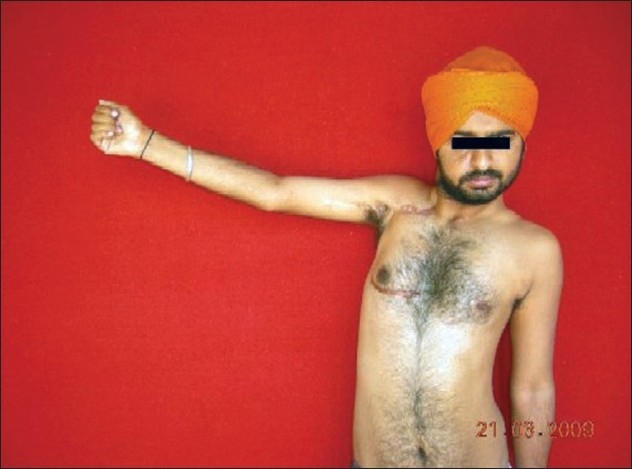
Shoulder abduction at 37 months

**Figure 7:**
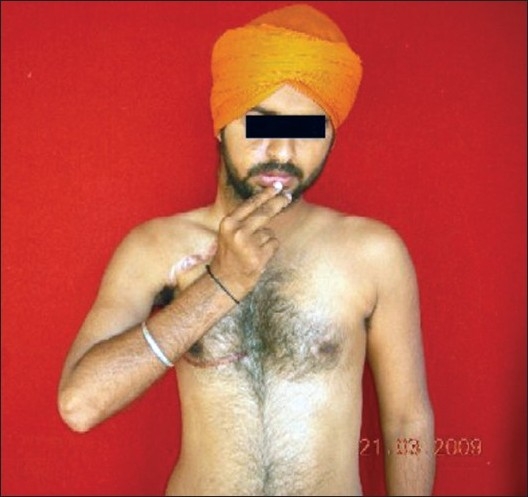
Elbow flexion at 37 months

**Figure 8:**
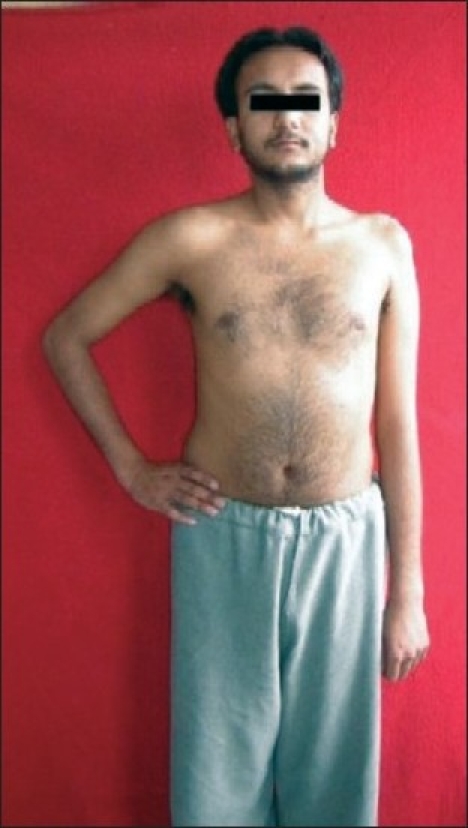
Left-sided upper brachial plexus palsy involving C5, C6 spinal nerves

**Figure 9:**
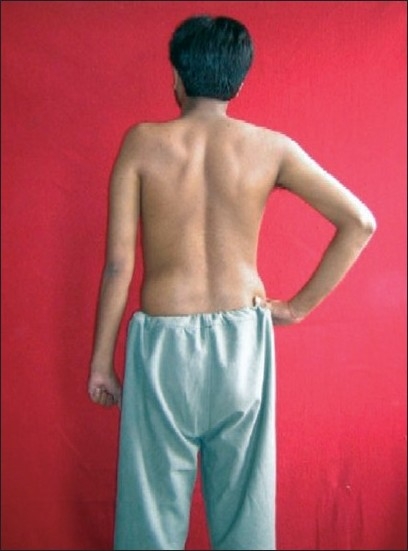
Wasting of supraspinatus, infraspinatus, deltoid & elbow flexors

**Figure 10:**
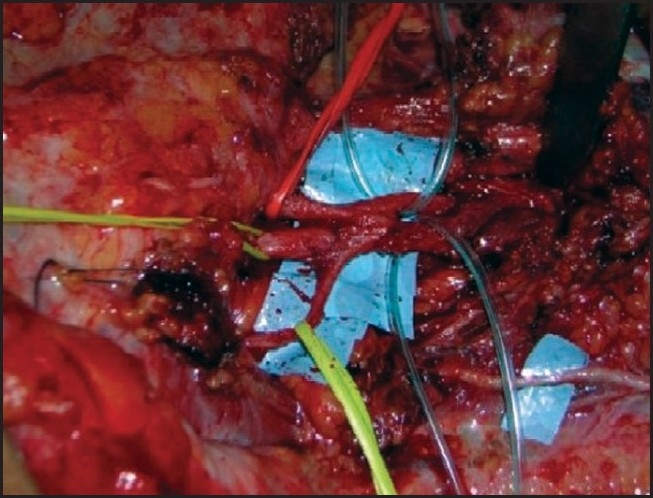
Extensive fibrosis in C5, C6 spinal nerves

**Figure 11:**
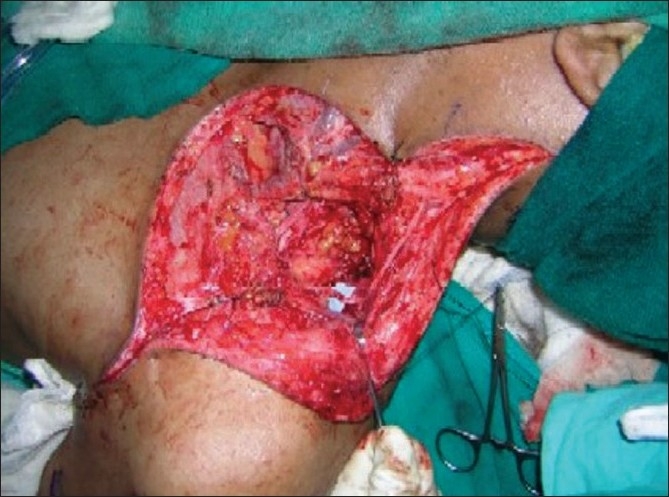
Transfer of spinal accessory nerve to suprascapular nerve

**Figure 12:**
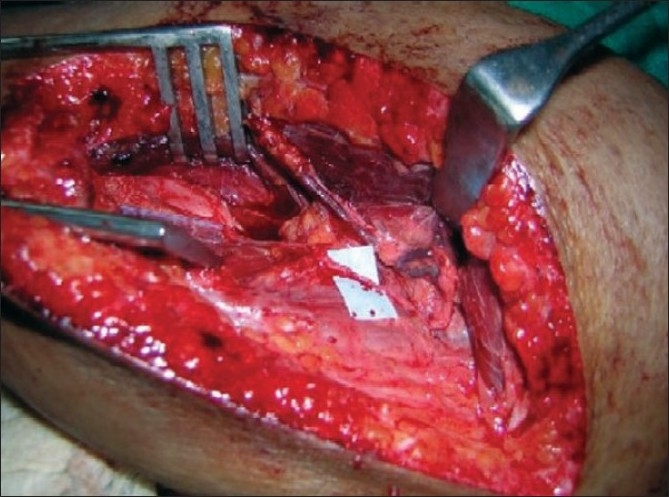
Motor branch to long head triceps muscle has been transfered to anterior branch of axillary nerve

**Figure 13:**
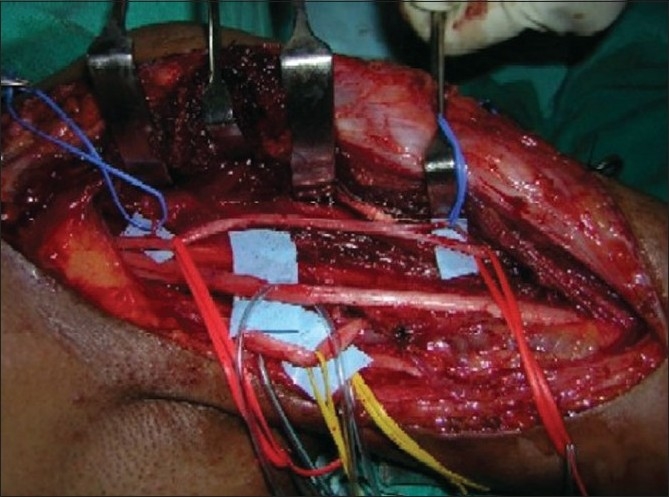
Motor branches to biceps and brachialis muscle are in blue tapes

**Figure 14:**
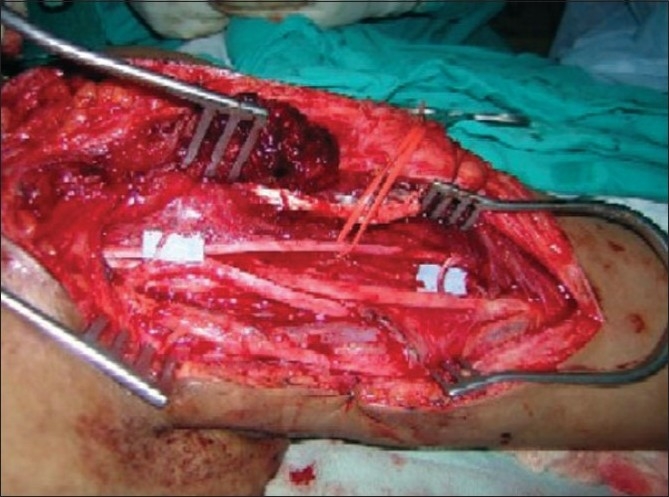
Proximally a single fascicle of ulnar nerve transferred to biceps motor branch (Oberlin I) & a median nerve fascicle transferred to brachialis motor branch distally(Oberlin II)

**Figure 15:**
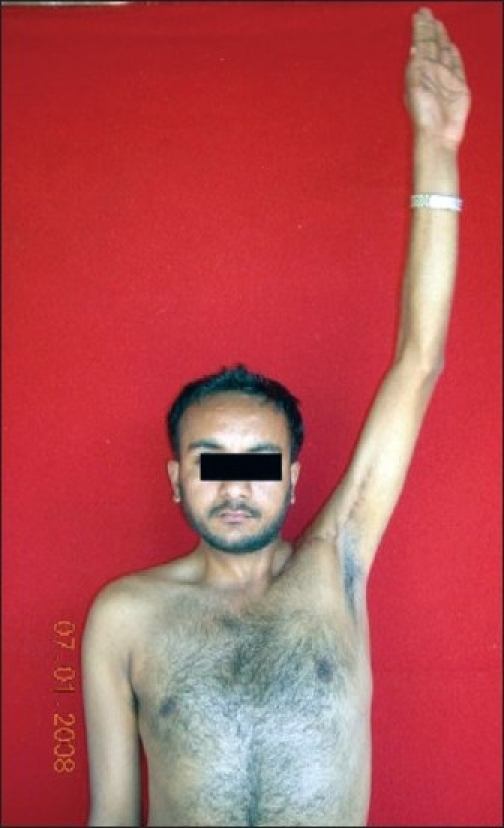
Patient restored 170 degree of shoulder abduction at 23 months

**Figure 16:**
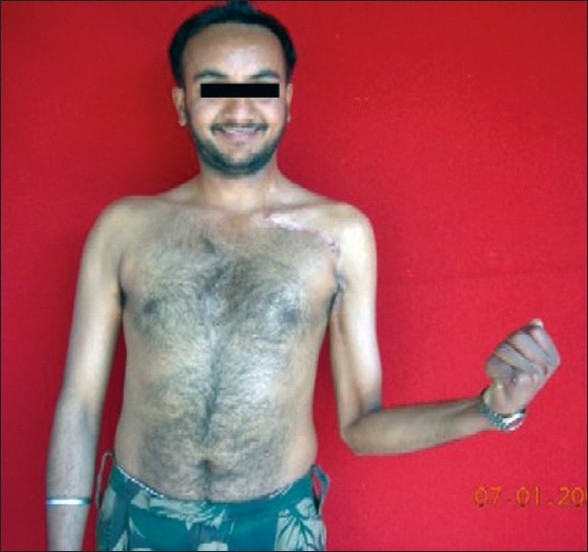
Contracting biceps at 23-month follow-up

20 patients with upper brachial plexus injury underwent microneuroreconstruction [Figures 1–16]. All the patients were male in the age group 20-41 years (mean age 25.7 years). The right brachial plexus was involved in 14 patients and the left plexus was involved in 6 patients. The mean period of time from injury to nerve reconstruction was 4.2 months (range: 3-10 months). In three patients, the brachial plexus lesion was associated with a clavicular fracture, and two had a rib fracture. High-velocity motorcycle accident was the most common cause of brachial plexus injury and accounted for injury in 15 patients [Table 4]. All patients had traction injuries.

**Table 4:** Type of accidents

*Type of accident*	*No. of patients*	*Percentage*
Motor cycle accident	15	75
Motor vehicle accident	3	15
Pedestrian	2	10

Two roots (C5 and C6) were involved in 12 patients and three roots (C5, C6 and C7) in 8 patients. In the presence of root avulsions, proximal root stumps were either not visible or some scarred nerve elements were seen. In other cases the scarred nerve roots, though in continuity, demonstrated either very weak or nil response on electric stimulation. Resection of these root stumps close to the intervertebral foramen showed fibrosis. Hence, nerve transfers were considered to restore shoulder and elbow functions. To restore shoulder abduction, distal spinal accessory nerve was transferred directly to the suprascapular nerve in 19 patients. In one patient, phrenic nerve was used as a donor for suprascapular nerve. In 11 patients, axillary nerve was also neurotized using different donors - radial nerve branch to the long head triceps (7 patients), intercostal nerves (2 patients) and phrenic nerve with nerve graft (2 patients).

To restore the elbow flexion, Oberlin transfers were performed in 14 patients. Three intercostal nerves (3rd, 4th and 5th) were transferred to the lateral part of musculocutaneous nerve in four patients. In one patient, spinal accessory nerve lengthened with a graft was transferred to the musculocutaneous nerve. In another patient, phrenic nerve with an interposed graft was used as a motor donor for musculocutaneous nerve. In four patients only Oberlin I procedure (transfer of ulnar nerve fascicle to the biceps motor branch) was performed. Double fascicular nerve transfers, i.e. Oberlin I and Oberlin II (transfer of median nerve fascicle to the brachialis motor branch), were performed in 10 patients. The study results are presented in Table 5.

**Table 5:** Clinical data

Patient no.	Age (years)	Sex	Interval between injury and surgery (months)	Period of follow-up (months)	Shoulder abduction (MRC grade)	Elbow flexion (MRC grade)
1	26	M	4	37	M4	M4
2	22	M	4	21	M3+	M3
3	30	M	6	22	M3	M4
4	34	M	3	29	M4	M4
5	21	M	10	19	M3	M3
6	26	M	4	20	M3	M3
7	33	M	3	23	M3	M3+
8	41	M	4	27	M4	M4
9	21	M	4	24	M3	M3+
10	28	M	3	28	M4	M4
11	20	M	3	28	M3	M3+
12	29	M	5	21	M3+	M4
13	22	M	3	19	M3	M3+
14	24	M	6	20	M3	M3+
15	21	M	4	26	M4	M4
16	20	M	3	18	M2+	M2+
17	22	M	3	20	M3	M3+
18	31	M	4	20	M3	M3
19	23	M	5	22	M3	M3+
20	21	M	3	21	M3+	M4

The minimum follow up period was 18 months in two patients.

Functions achieved were assessed as poor to excellent. In shoulder abduction, five patients scored M4 [Figure 15] and three patients M3+. Fair results were obtained in rest of 12 patients. Degree of abduction averaged 95° (range, 50-170°). Useful external rotation was restored in 13 patients. Eight patients scored M4 in elbow flexion [Figures 7,16] and assessed as excellent results. Good results (M3+) were obtained in seven patients. Five patients had fair results (M2+ to M3). In Oberlin transfer, the mean period of time from surgery to electromyography recovery of the biceps and brachialis muscles was 2.5 months (range: 2-5) and 2.8 months (range: 2-5), respectively. In intercostal to musculocutaneous nerve transfer, the EMG signs of biceps reinnervation appeared quite late (average 10.5 months, range: 8.5-12 months). With spinal accessory nerve and phrenic nerve transfers, the initial reinnervation potentials were recorded at 8 and 8.5 months, respectively.

Four patients in the Oberlin group experienced transient paresthaesia in little finger (Oberlin I) and two in the index finger (Oberlin II) that lasted for 2-3 weeks. Three patients in Oberlin II group developed pointing index which improved in 6-10 weeks. Use of a single fascicle of ulnar or median nerve did not significantly affect the hand functions. Patients with excellent results could lift 3.5 kg of weight. Extension of elbow was downgraded from M4 to M3 in four patients who had undergone transfer of long-head branch of triceps to anterior branch of axillary nerve.

Use of phrenic nerve consistently produced ipsilateral diaphragmatic palsy, though all the four patients were asymptomatic. Use of three intercostal nerves did not affect the pulmonary functions.

Younger patients (<25 years), short denervation period (<4 months), direct transfers (without a graft), combined transfers (more than one donor nerve to restore a single function), intraplexal transfers (Oberlin I and II procedures) and transfers close to the motor end plates of the target muscles achieved consistently good results. Patients with C5 and C6 injuries restored better functions than those with an associated C7 injury.

## DISCUSSION

Upper brachial plexus injuries account for 15-20% of supraclavicular plexus injuries.[10] Repair of these injuries offer good prognosis because the hand functions are preserved. In root avulsions, nerve repair is not possible, and nerve transfers offer far superior results over tendon/muscle transfers or shoulder arthrodesis.[11] Also in some upper plexal injuries, extensive fibrosis make the proximal root stumps of doubtful viability. In such cases, nerve transfer remains the only viable option of rehabilitating the arm. It is well accepted that the two main priorities in nerve transfers are the restoration of elbow flexion and shoulder abduction. Elbow flexion has been achieved with many donor nerves including the intercostal nerves,[6,7,11,12–16] medial pectoral nerve,[5,17] phrenic nerve,[3,4] thoracodorsal nerve,[18] spinal accessory nerve[1,2] and recently introduced Oberlin transfer.[8] An intercostal nerve contains no more than 500 motor fibres;[15] hence, at least two or three intercostals nerves (T3, T4 and T5) are coapted with the motor component of musculocutaneous nerve. Some surgeons do not recommend intercostals to musculocutaneous transfer[19] as the surgery is challenging and time consuming,[20] results are not consistent,[21] and life-threatening complications have been observed.[22]

Transfer of medial pectoral nerve to the musculocutaneous nerve is one of the most controversial procedures.[5] In 1948, Lurje[23] described the use of this nerve as a donor in patients with Erb's palsy. Thereafter, only a few reports of the use of this nerve transfer were published. Some authors do not recommend this type of nerve transfer at all.[5] Chuang *et al*.[11] and Gu *et al*.[3,4] have popularized the transfer of phrenic nerve to musculocutaneous nerve (either directly or with a sural nerve graft). This procedure again has not gained wide acceptance amongst the western surgeons as it sacrifices an important motor nerve, contraindicated in children and cannot be combined with simultaneous intercostal nerve transfer.[20] The spinal accessory nerve has the disadvantage of requiring a long nerve graft to reach the musculocutaneous nerve.[24]

Transfers of a single fascicle of ulnar nerve to the motor branch of biceps[8] and a median nerve fascicle to the brachialis[25] have produced the most promising results, as there is no wastage of any donor nerve fibres to the sensory part of musculocutaneous nerve. Since the nerve transfer is performed close to the target muscle, the return of function is faster. This technique requires no special re-education of the muscle. Sparing of 1 or 2 fascicle forming the ulnar and median nerves does not result in any subjective deficit of hand function.[25,26] Preoperative and postoperative evaluations of pinch strength, grip strength and two point discrimination at the pulp of little and index fingers remain unaltered. In the series reported by Somsak Leechavengvongs *et al*.,[26] 32 patients with absent elbow flexion underwent nerve transfer using 1 or 2 fascicles of the ulnar nerve to the motor branch of the biceps muscle. Twenty-six patients had root avulsion injury of C5 and C6; 4 had root avulsion injury of C5, C6 and C7; and 2 had lateral and posterior cord injury with distal injury of the musculocutaneous nerve. The mean denervation period was 6 months. At the final follow-up, 30 patients had biceps strength of M4 and 1 had biceps strength of M3. One elderly patient operated 1 year after injury did not demonstrate any sign of recovery. In Mackinnon series,[27] six patients underwent double fascicular transfers. At the final follow-up evaluation, elbow flexion strength was MRC grade 4+ in four patients and grade 4 in two patients. In the series reported by Liverneaux *et al*.,[28] 15 patients underwent double nerve transfers to restore elbow flexion. The authors had a follow-up of more than 6 months in 10 of them. Six had C5, C6 injuries, three had C5, C6 C7 palsies and one had sustained an infraclavicular injury. The average delay before surgery was 6.6 months. Grade 4 elbow flexion was restored in each of the 10 patients. In Sungpet series,[29] 36 patients with upper root avulsions underwent transfer of a single fascicle from the ulnar nerve to the proximal motor branch of the biceps muscle. Thirty- four patients achieved biceps strength of MRC grade 3 or better. Importantly, they also included two patients with C5, C6 and C7 avulsions. All these studies highlight the reliability of fascicular transfers in restoring elbow flexion in upper brachial plexus injuries.

Shoulder stability and abduction can be restored by arthrodesis, muscle tendon transfer and nerve transfers. Shoulder arthrodesis yields a poor range of motion.[11] It is difficult to achieve satisfactory abduction by muscle/ tendon transfers with use of trapezius, levater scapulae, sternocleidomastoid or latissimus dorsi muscles.[11,30,31] Nerve transfer, however, provides good range of shoulder abduction and stability.[32,33] Transfer of distal spinal accessory nerve to the suprascapular nerve restores an average of 45° of abduction and some external rotation by reactivating the supraspinatous and infraspinatous muscles. A simultaneous transfer of suprascapular nerve and axillary nerve yields much better results when adequate donors are available.[11,34,35] Axillary nerve neurotization can be performed through an anterior approach using phrenic nerve, distal spinal accessory nerve, intercostal nerves or medial pectoral nerve as donor nerves. This approach not only requires nerve grafts but also results in dilution of nerve fibres entering the deltoid muscle.[36] A posterior approach allows the transfer of nerve to the long head of triceps (which contains mainly motor fibres) to the anterior branch of axillary nerve which innervates the anterior and middle deltoid muscles. This transfer avoids the misdirection of the regenerated axons into the cutaneous branch and teres minor.[37] The functional loss is minimal and is compensated by remaining two heads of triceps and the teres muscle group. Amongst the three heads of the triceps, the long head plays the least important role during elbow extension.[38] The long head of triceps has been transferred for axillary contracture[39] and as a free functioning muscle transfer.[40] Leechavengvong *et al*.[41] reported seven patients with C5 and C6 avulsion injuries who underwent double nerve transfers (distal spinal accessory nerve to suprascapular nerve and long head triceps branch to anterior branch of axillary nerve). The mean shoulder abduction achieved was 124°. Same authors[42] report a series of 15 patients with 8 patients achieving shoulder abduction in the range of 130°-160°. Again in all these patients, root avulsions were confirmed prior to reconstruction.

Complete avulsion of all roots of the brachial plexus is a serious problem. In the 1980s, several workers[43,44] observed in animal models that implantation of a peripheral nerve graft into the spinal cord can induce regeneration of spinal motor neurons. Carlstedt[45] was the first to apply these observations in human beings. He treated a patient with C6 to T1 root avulsion injury by implanting two ventral roots into the spinal cord through slits in the piamater, C6 directly and C7 via a nerve graft. At 3 years power in biceps was M4 and patient had voluntary activity also in the deltoid (M2), triceps (M1-2) and brachioradialis (M1-2). In 2000, he and others[46] published the results of the reimplantation technique in a larger group of patients. Bertelli *et al*.[47] noticed the improvement in proximal muscle function and opined that this improvement is limited and does not justify the use of spinal implants. In 2005, Fournier *et al*.[48] concluded that the outcomes of root implantation were modest and results were better when the diagnosis of avulsion was made within 10 days and reparative surgery undertaken within 3 weeks of injury.

In our series of patients, combined transfers (Oberlin I and II) produced better results than single (Oberlin I). We believe that in upper root injuries, the quantity of functioning motor axons in the ulnar nerve fascicle may not be adequate in some cases (e.g. those with C5, C6 and C7 spinal nerve injuries). This may account the need for additional procedures such as Steindler flexorplasty. Brachialis muscle is a strong elbow flexor and its innervation along with biceps muscle gives a stronger elbow flexion. Use of ipsilateral C7, as a source of motor axons, is not favoured by most of the workers. Chuang[49] prefers connecting the ipsilateral C7 nerve fibres to distal C7 only because in his opinion the transfer of C7 in restoration of critical function provides unreliable results. In our opinion, when completely intact, it is probably safest to leave the middle trunk undisturbed and instead select branches from the posterior cord (triceps motor branches) and/or extraplexal nerves (i.e. spinal accessory nerve, or intercostal nerves) as possible donors.

Our indications for nerve transfers have been root avulsion injuries as well as extensive fibrosis in the proximal plexus with possible intact long thoracic and dorsal scapular nerves. Instead of dissecting very proximally or performing laminectomies, we favoured distal nerve transfers. Even in the presence of rudimentary viable root stumps, nerve grafting would have been technically difficult in these cases.

We observed that in some patients with C5, C6 and C7 injuries, the intraplexal fascicular transfer might not be suitable because of weakness of triceps and the variable contribution of C7 fibres in the formation of ulnar and median nerves. In these patients, we have used extraplexal donor nerves (e.g. spinal accessory nerve, intercostal nerves and phrenic nerve).

In the management of upper brachial plexus injuries, intraplexal nerve transfers produce better results than extraplexal transfers. The other favourable factors are younger patients with short denervation period, direct transfers, use of multiple donor nerves to restore a single function and selective neurotization close to the target muscles.
